# Cross-Sectional Study of Q Fever Seroprevalence among Blood Donors, Israel, 2021

**DOI:** 10.3201/eid3005.230645

**Published:** 2024-05

**Authors:** Nesrin Ghanem-Zoubi, Yafit Atiya-Nasagi, Evgeniy Stoyanov, Moran Szwarcwort, Basel Darawsha, Mical Paul, Eilat Shinar

**Affiliations:** Rambam Health Care Campus, Haifa, Israel (N. Ghanem-Zoubi, M. Szwarcwort, M. Paul);; The Ruth and Bruce Rappaport Faculty of Medicine, Technion-Israel Institute of Technology, Haifa (N. Ghanem-Zoubi, B. Darawsha, M. Paul);; Israel Institute for Biological Research, Ness-Ziona, Israel (Y. Atiya-Nasagi);; Magen David Adom National Blood Services, Ramat Gan, Israel (E. Stoyanov, E. Shinar)

**Keywords:** Q fever, zoonoses, bacteria, *Coxiella burnetii*, seroprevalence, blood donors, Israel

## Abstract

We evaluated Q fever prevalence in blood donors and assessed the epidemiologic features of the disease in Israel in 2021. We tested serum samples for *Coxeilla burnetii* phase I and II IgG using immunofluorescent assay, defining a result of >200 as seropositive. We compared geographic and demographic data. We included 1,473 participants; 188 (12.7%) were seropositive. The calculated sex- and age-adjusted national seroprevalence was 13.9% (95% CI 12.2%–15.7%). Male sex and age were independently associated with seropositivity (odds ratio [OR] 1.6, 95% CI 1.1–2.2; p = 0.005 for male sex; OR 1.2, 95% CI 1.01–1.03; p<0.001 for age). Residence in the coastal plain was independently associated with seropositivity for Q fever (OR 1.6, 95% CI 1.2–2.3; p<0.001); residence in rural and farming regions was not. Q fever is highly prevalent in Israel. The unexpected spatial distribution in the nonrural coastal plain suggests an unrecognized mode of transmission.

Q fever, caused by infection with the bacterium *Coxeilla burnetii*, is an endemic disease in Israel. In 2021, a total of 341 cases of Q fever were reported to the Epidemiology Division, Ministry of Health (*1*), representing an estimated incidence of 3.6/100,000 population. For comparison, the highest incidence of Q fever in recent years in the European Union was reported from Spain, reaching 0.7/100,000 population in 2019 (*2*). Despite the longstanding endemicity of Q fever in Israel, little is known about the actual epidemiology, geographic distribution, and groups at highest risk for the disease (*3*–*6*). Ministry of Health data are based on passive notifications, relying on reporting by the treating physician and laboratory staff where the diagnostic test suggestive of Q fever was performed. This reporting system is known to be highly affected by the awareness and motivation of medical staff (both physicians and laboratory workers). Almost half of acute Q fever cases are asymptomatic (*7*), but infections can progress years later to the more severe, chronic form of the disease. Thus, the actual population incidence of Q fever, including events missed by the passive surveillance system, has not been documented in Israel. In this study, we evaluated the prevalence of Q fever in adult healthy volunteer blood donors in Israel and assessed the epidemiologic features of the disease.

## Methods

### Study Design and Setting

We conducted a cross-sectional nationwide prevalence study using blood samples collected during January 1–October 8, 2021. Samples were collected by Magen David Adom (MDA) National Blood Service. Blood donations were performed in locations across the country. Samples were collected and stored from donors who signed an informed consent agreeing to participate in the study. Participants completed an obligatory donor health questionnaire that included their demographic details and health status. The study was performed in line with the principles of the Declaration of Helsinki. The study was approved by the ethics committees of Rambam Health Care Campus and MDA’s research committee. Informed consent was obtained from all individual participants included in the study.

### Participants

Participants were male and nonpregnant female adult blood donors >18 years of age (pregnant women are excluded from blood donation) who completed the donor health questionnaire and signed the MDA consent form for use of blood donations in research. Samples for this study were selected randomly from the full sample pool (≈1,000 donations/day) by donation site from all districts in Israel. To achieve a study group representative of the whole population, we enriched the final study set with samples taken from female donors and from Arab-populated locations; however, sample selection was performed randomly from the respective sample pools.

### Study Flow and Data Collection

We centrifuged specimens obtained from blood donors, saved serum samples at 4°C, and transferred samples to the reference laboratory for antibody testing. We kept samples anonymous and did not convey results to the blood donors.

We collected demographic data on date of donation, date and country of birth, sex, and location of residence. We extracted data on the residence localities’ sociodemographic characteristics from the Israeli Central Bureau of Statistics (CBS) website. Those data consisted of population size, ethnicity of population, and socioeconomic index cluster (categorized as 1–10 on the basis of multiple social and economic variables). We defined Arab localities as localities in which >80% of the population were Arab. Localities were classified by socioeconomic cluster as low (1–3), middle (4–7), or high (8–10) status. Rural localities were defined as those with <5,000 inhabitants.

### Microbiological Methods

We performed screening for presence of phase I and phase II *C. burnetii* IgG by using an in-house indirect immunofluorescence assay at the National Central Laboratory for Rickettsiosis in the Israel Institute for Biologic Research. We performed the test by using antigens of phase I Ohio strain and phase II Nine Mile strain of *Coxiella burnetii*. This test has been used clinically for many years as the standard for Q fever diagnosis in Israel. The test was externally validated upon its introduction and continues to be under periodic internal and external quality assurance control programs. Results were reported as borderline if positive in a titer of 100 and seropositive with antibody titers of phase I or phase II IgG >200.

### Seroprevalence Spatial Distribution

We calculated Q fever seroprevalence rate by locality, natural region, and subdistrict and district level. Israel is divided into districts and subdistricts on the basis of administrative considerations and into natural regions on the basis of regional geographic features. We used the natural regions to divide the country into 4 areas: the coastal plain and the inner regions of the north, center, and south. We created prevalence choropleth maps according to district, subdistrict, and natural region classification. We created maps using ArcGIS Desktop software (Esri, https://www.esri.com) to display spatial prevalence data.

### Statistical Methods

We expressed seroprevalence rates as an overall rate and in geographic and demographic groups. We calculated the sex- and age-adjusted seroprevalence to estimate national Q fever prevalence in adults >18 years of age using the sex and age distribution of the population of Israel according to 2019 CBS data. We used the χ^2^ test to assess the association between available epidemiologic characteristics and seropositivity for Q fever. We used binary logistic regression for multivariate analysis. We included variables with p<0.05 in bivariate analysis in the binary regression. All statistical tests were 2-tailed.

The sample size needed for prevalence estimation was calculated targeting a 95% CI and assuming a Q fever prevalence in Israel of 4% (on the basis of previous studies from Q fever–endemic developed countries that reported a prevalence range of 3%–5%). We targeted a precision of 1%. The sample size needed was calculated to be 1,476.

## Results

During January 1–October 8, 2021, samples were collected from 1,600 blood donors; we tested 1,473 samples with complete epidemiologic data. Of those, 188 (12.7%) samples were positive for *C. burnetii* antibodies. Phase II IgG >200 was detected in 187 participants; concomitant phase I IgG >200 was detected in 57 (30%) of those participants’ samples. Only 1 sample was found with positive phase I IgG and negative phase II IgG (Table 1). 

**Table 1:** Q fever antibody results in cross-sectional study of Q fever seroprevalence among blood donors, Israel, 2021

Antibody	No. (%) participants				
	Negative	Borderline, = 100	Positive, >200	200–800	>1,600
Phase II IgG	1,152 (78.2)	134 (9)	187 (12.6)	158 (10.7)	29 (1.9)
Phase I IgG	1,374 (93.2)	41 (2.7)	58 (3.9)	56 (3.8)	2 (0.1)

The mean age of the participants was 36.6 + 13.9 years; 859 (58.3%) were men and 614 (41.7%) women. The sex- and age-adjusted prevalence rate was 13.9% (95% CI 12.2%–15.7%).

The seroprevalence of Q fever was higher among men (14.9% [128/859]) than women (9.8% [60/614]) (Table 2). In addition, seroprevalence increased with age, reaching a rate of 17.4% (52/299) among participants >50 years of age. Participants living in rural locations had seropositivity of 10.6% (51/480). Seroprevalence rates were similar in Arab and Jewish localities and in low, middle, and high socioeconomic status localities.

**Table 2:** Crude prevalence of anti*-Coxiella burnetti* IgG in cohort groups in cross-sectional study of Q fever seroprevalence among blood donors, Israel, 2021

Parameter	No. participants	No. seropositive (%)
Overall	1,473	188 (12.7)
Sex		
M	859	128 (14.9)
F	614	60 (9.8)
Age group, y		
18–30 y	550	50 (9.0)
31–50 y	502	85 (16.9)
>50 y	299	52 (17.4)
Geographic factors		
Born in Israel	1,205	153 (12.7)
Living in Jewish locality	1,310	167 (12.7)
Living in Arab locality	157	21 (13.4)
Rural	372	38 (10.2)
Urban	1,095	150 (13.7)
Socioeconomic status of residence localities		
Low	328	39 (11.9)
Middle	714	94 (13.2)
High	416	54 (13.0)

Seropositive cases were widely distributed across all parts of the country. Prevalence rates varied among regions; rates were highest in the coastal plain (15.9% [100/629]) and lowest in the noncoastal central region (8.8% [37/419]) (Figure; Appendix Table 1). 

**Figure:**
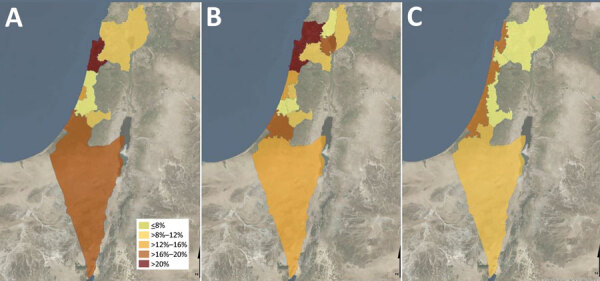
Q fever prevalence by district, subdistrict, and natural region in cross-sectional study of seroprevalence among blood donors, Israel, 2021. Spatial distribution of Q fever seroprevalence uses different geographic classifications. A) Seroprevalence rates by district; the highest rate was in Haifa district. B) Seroprevalence rates by subdistrict; the highest rate was in Hadera subdistrict. C) Seroprevalence rates by 4 natural region clusters; the highest rate was in the coastal plain area.

In bivariate comparison, older age, male sex, and residence in the coastal plain were more prevalent in the seropositive group than the seronegative one (Table 3). Residence in a rural area was less prevalent in the seropositive group but did reach statistical significance. In multivariate logistic regression analysis, variables that were independently associated with Q fever seropositivity were age (odds ratio [OR] 1.2, 95% CI 1.01–1.03; p<0.001), male sex (OR 1.6, 95% CI 1.1–2.2; p = 0.005), and residence in the coastal plain (OR 1.6, 95% CI 1.2–2.3; p<0.001).

**Table 3:** Risk factors for Q fever seropositivity and results of univariate and multivariate analyses in cross-sectional study of Q fever seroprevalence among blood donors, Israel, 2021*

Parameter	All participants, N = 1,473	Seropositive, n = 188	Seronegative, n = 1,285	Univariate analysis p value	Multivariate analysis	
					Odds ratio (95% CI)	p value
Mean age +SD, y	36.6 +13.9	41.1 +13.3	35.9 +13.9	<0.001	1.2 (1.01–1.03)	<0.001
Male sex	859 (58.3)	128 (68.1)	731 (56.9)	0.004	1.6 (1.1–2.2)	0.005
Born in Israel	1,211 (82.9)	153 (81.3)	1,058 (83)	0.677		
Living in Jewish locality	1,310 (89.3)	167 (88.8)	1,143 (89.4)	0.824		
Rural locality	372 (25.4)	38 (20.2)	334 (26.1)	0.082		
Socioeconomic status of residence localities				0.683		
Low	328 (22.5)	39 (20.9)	289 (22.7)			
Moderate	714 (49.0)	94 (50.3)	620 (48.8)			
High	416 (28.5)	54 (28.9)	362 (28.5)			
Living in coastal plain	629 (42.9)	100 (53.2)	529 (41.4)	0.002	1.6 (1.2–2.3)	<0.001

*Values are no. (%) except as indicated. †<5,000 population.

## Discussion

We found high Q fever seroprevalence at an estimated rate of 13.9% among healthy adults in Israel, with wide geographic distribution. Seropositive status was independently associated with male sex, older age, and residence in the coastal plain.

National seroprevalence studies of Q fever from the past 2 decades have been conducted in several countries. In the United States (*8*), the Netherlands (before the large outbreak in 2007) (*9*), and Chile (*10*), prevalence was ≈3%. Higher prevalence rates were reported from Australia (5.6%) (*11*), French Guiana (9.6%) (*12*), and Northern Ireland (12.8%) (*13*). Smaller studies from other countries demonstrated a broad range of Q fever prevalence: 6.9% in Bhutan (*14*), 6.9% in Reunion Islands (*15*), 24.2% in Jordan (*16*), and 52.7% in Cyprus (*17*). Those studies used different testing methods and different cutoff titers to define seropositivity, which could have contributed to differences in reported prevalence rates. 

In this study, we used a relatively high cutoff titer to define seropositivity, which could have led to an underestimation of seroprevalence rate. For example, if we had used a cutoff of 100 instead of 200 to define seropositivity, the crude overall prevalence rate would have increased from 12.7% to 21.8%. Nevertheless, the high seroprevalence rate we found seems to correlate with reported ongoing and increasing incidence of the disease, as reflected in clinical reports in the national surveillance (*1*) (Appendix Figure). The increase in incidence observed in recent years had been preceded by several outbreaks reported in different areas (*18*–*20*).

Increasing age and male sex as risk factors for Q fever have been reported in various countries in both clinical and epidemiological studies (*8*,*11*,*21*,*22*); possible explanations have been related to increased exposure from living in endemic places and certain occupational exposures. We did not have occupational data for participants in this study, but previous local reports have frequently mentioned that animal exposure does not seem to be a risk factor (*23*–*25*). Similarly, residence in a rural area was not associated with Q fever seroprevalence in our study, contrary to previous reports (*11*,*15*,*17*,*26*). Yet, the significance of male sex as a risk factor might be related to exposure factors that should be further investigated.

Residence in the coastal plain was significantly associated with Q fever prevalence in our study. Of note, the coastal plain region in Israel contains more urban and highly populated localities than other regions, whereas livestock farms are more prevalent in noncoastal areas, mainly in the south and north (*27*). For example, the subdistrict with the highest seroprevalence rate in our study was Hadera (26%); only 7.2% of sheep farms and 4.2% of cattle farms in Israel are located in that subdistrict (*27*). Wind can carry sporelike forms of *C. burnetii* to distances of up to 18 km, as documented in previous studies (*28*,*29*). The short 17-km distance of Hadera subdistrict from the eastern border (an area for which data are missing) should be taken into consideration in further studies. However, those factors alone cannot explain the unique spatial pattern of prevalence depicted in our study; in those circumstances, we would have expected a decreasing gradient of prevalence in which the highest rates would be observed in rural and inner noncoastal regions. Our spatial prevalence data point to an unknown mode of transmission of the disease that seems to play a major role in current epidemiology in Israel. The possibility of an unrecognized animal that might be a reservoir for the bacterium in the urban environment should be investigated. Special attention should be paid to stray cats and wild boars, particularly because wild boars have become a frequent observation inside Haifa, the largest city in the northern coastal plain. In addition, rivers and streams could theoretically play a role in the transmission of the bacterium from the inner areas of the country to the coastal plain; in 1 study, *C. burnetii* was detected in contaminated river water (*30*).

Compared with brucellosis, another endemic zoonotic disease in Israel, in which most cases occur among Arabs (*31*), in this study, Q fever seems to affect Arab and Jewish locations at similar rates. The transmission mode of brucellosis is clearly associated with consumption of unpasteurized dairy products and direct contact with infected sheep (*32*). The fact that sheep herds, which are most likely the common source of both diseases, are found mostly inside or in close proximity to Arab locations (*27*) supports a different transmission mode of Q fever.

The first limitation of our study is the epidemiologic differences between healthy blood donors and the general population. We did not include persons <18 years of age, pregnant women, and persons who were not qualified to donate blood because of responses on the donor health questionnaire or results of physical tests performed before donation (i.e., blood pressure, heart rate, hemoglobin levels). For estimating national prevalence, we adjusted our results to age and sex distribution of the general population. Still, underrepresentation of Arab population in our cohort was prominent and might have missed prevalence ethnic differences. Another limitation is the absence of data on individual risk factors such as occupation and animal exposure. As mentioned previously, those data were not found to be associated with clinical illness in reports from Israel (*23*) but should be further studied prospectively.

In conclusion, our study presents key data on the epidemiology of Q fever in Israel, demonstrating high seroprevalence throughout the country, with predominance in the coastal plain for unknown reasons and no association with rural areas or livestock farms. Future studies should further investigate environmental factors that seem to play a major role in the transmission of this endemic disease. Improving our knowledge of disease transmission is critical in planning prevention programs for this highly endemic disease with significant consequences for public health. 

Appendix Additional information about cross-sectional study of Q fever seroprevalence among blood donors, Israel, 2021
