# Supplementary material for: Cross-Sectional Study of Q Fever Seroprevalence among Blood Donors, Israel, 2021
Source: Emerg Infect Dis. 2024 May;30(5):941–6. doi: 10.3201/eid3005.230645 (PMC11060453; doi:10.3201/eid3005.230645)
Supplement: Appendix — Additional information about cross-sectional study of Q fever seroprevalence among blood donors, Israel, 2021 [file 23-0645-Techapp-s1.pdf]

Article DOI: https://doi.org/10.3201/eid3005.230645

*EID cannot ensure accessibility for supplementary materials supplied by authors. Readers who have difficulty accessing supplementary content should contact the authors for assistance.*

# Cross-Sectional Study of Q Fever Seroprevalence among Blood Donors, Israel, 2021

## Appendix

**Appendix Table.** Seroprevalence of Q fever according to spatial classification by district, subdistrict, and natural regions

| Area | No. participants | No. seropositive (%) | Area | No. participants | No. seropositive (%) |
|---|---|---|---|---|---|
| District | | | Sub-district | | |
| North | 355 | 40 (11.3) | Golan | 125 | 13 (10.4) |
| | | | Tzfat | 82 | 5 (6.1) |
| | | | Akko | 29 | 6 (20.7) |
| | | | Kinneret | 36 | 7 (19.4) |
| | | | Yizre'el | 83 | 9 (10.8) |
| Haifa | 141 | 31 (22) | Haifa | 107 | 22 (18.7) |
| | | | Hadera | 34 | 9 (26.4) |
| Center | 278 | 22 (7.9) | Ramla | 52 | 2 (3.8) |
| | | | Sharon | 78 | 11 (14.1) |
| | | | Petah Tikva | 62 | 5 (8) |
| | | | Rehovot | 86 | 4 (4.6) |
| Tel Aviv | 177 | 28 (15.8) | Tel Aviv | 177 | 28 (15.8) |
| Jerusalem | 181 | 19 (10.5) | Jerusalem | 181 | 19 (10.5) |
| South | 234 | 39 (16.6) | Ashkelon | 170 | 31 (18.2) |
| | | | Be'er-Sheva | 64 | 8 (12.5) |
| Natural region | | | | | |
| Coast plain | 629 | 100 (15.9) | | | |
| Noncoastal north | 347 | 40 (11.5) | | | |
| Noncoastal center | 419 | 37 (8.8) | | | |
| Noncoastal south | 86 | 12 (13.9) | | | |

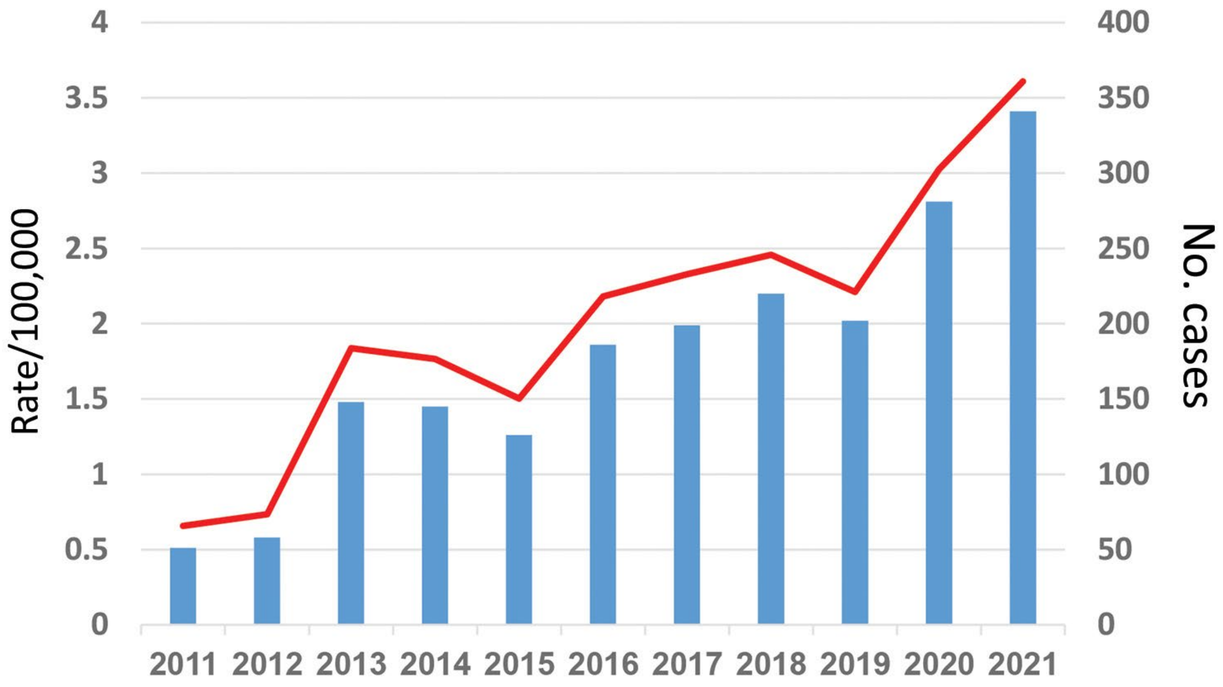


**Appendix Figure.** Q fever incidence in Israel during 2011–2021. Calculated incidence of Q fever in Israel in the years 2011 to 2021. Number of annual Q fever cases according to data from Division of Epidemiology of Ministry of Health and annual population data according to Israeli Center Bureau of Statistics. As a mandatory notifiable disease, each new case of Q fever must be reported to Ministry of Health. Data are published in a weekly report of all mandatory notifiable diseases (https://www.gov.il/he/Departments/DynamicCollectors/weekly-epidemiological-report).
